# Predictive value of ^18^F-FDG PET/CT PyRadiomics features using a tabular deep learning model for cancer-related cognitive impairment in patients with DLBCL without CNS involvement

**DOI:** 10.3389/fonc.2026.1861773

**Published:** 2026-08-12

**Authors:** Jing Wang, Yiyuan Dong, Ningjun Xu, Xiaowei Ma, Ying Liu, Qian Zhao

**Affiliations:** 1 General Hospital of Ningxia Medical University, Yinchuan, Ningxia Hui Autonomous Region, China; 2 Division of Chinese Medicine, School of Pharmacy, Macau University of Science and Technology, Macau, Macau SAR, China

**Keywords:** 18F-fluorodeoxyglucose, cancer-related cognitive impairment, diffuse large B-cell lymphoma, PyRadiomics features, SHAP, tabular deep learning

## Abstract

**Objective:**

To evaluate the predictive value of tabular deep learning models trained on handcrafted ^18^F-FDG PET/CT PyRadiomics features and clinical data for cancer-related cognitive impairment (CRCI) among patients with diffuse large B-cell lymphoma without central nervous system involvement (non-CNS DLBCL).

**Material and methods:**

We retrospectively analyzed 112 non-CNS DLBCL patients who underwent brain ^18^F-fluorodeoxyglucose (^18^F-FDG)PET/CT. Based on Montreal Cognitive Assessment (MoCA) and Functional Assessment of Cancer Therapy-Cognitive Function (FACT-Cog) scores, patients were classified into the CRCI group (n = 73) and the non-CRCI group (n = 39) and randomly partitioned into training (n=89) and test (n=23) sets at an 8:2 ratio. Five-fold cross-validation was performed within the training set for model development, and bootstrap resampling was used to estimate 95% confidence intervals for AUC. Radiomic features were extracted with PyRadiomics from automatically segmented brain regions using MOOSE120 software. These handcrafted PET/CT radiomic features and clinical variables were organized as structured tabular datasets; raw PET/CT images were not used as direct model inputs. Feature selection was performed using correlation coefficients. The performance of seven tabular deep learning models was evaluated using receiver operating characteristic (ROC) curves and decision curve analysis (DCA), and different input modalities (clinical, PET, CT, and combined) were compared. Shapley additive explanations (SHAP) were applied to interpret feature contributions.

**Results:**

The AutoInt and Neural Oblivious Decision Ensembles (NODE) models showed the best predictive performance, achieving training areas under the curve (AUCs) of 0.905 and 0.904, and test AUCs of 0.858 and 0.808, respectively. DCA suggested potential clinical utility. In the modality-specific analysis, the AutoInt model integrating CT, PET, and clinical features achieved a training AUC of 0.905 and a test AUC of 0.858, representing the most favorable test-set performance among the evaluated AutoInt input modalities. A CT texture feature from the left occipital lobe emerged as the highest-ranked model contributor in SHAP analysis, with a mean absolute attribution score of approximately 0.0078.

**Conclusions:**

Tabular deep learning models based on handcrafted PET/CT PyRadiomics features showed preliminary potential for predicting CRCI in patients with non-CNS DLBCL. Among the evaluated models, AutoInt achieved the most favorable performance in the exploratory internal test set. Multimodal integration of PET, CT, and clinical tabular features may provide complementary information for CRCI prediction. SHAP analysis identified a left occipital CT texture feature as the top-ranked model contributor; however, this finding should be regarded as exploratory rather than a validated biomarker.

## 1 Introduction

Diffuse large B-cell lymphoma (DLBCL) is the most common subtype of non-Hodgkin lymphoma and represents an aggressive but potentially curable hematological malignancy (1, 2). The R-CHOP regimen has significantly improved survival outcomes in DLBCL patients; however, approximately 20%–50% of cases still develop refractory disease or experience relapse. Recent years have witnessed substantial therapeutic advances, including novel cytotoxic agents, optimized stem cell transplantation techniques, and targeted immunotherapies such as BCL-2 and PD-1 inhibitors. These developments have greatly expanded treatment options for patients with refractory DLBCL, thus improving clinical outcomes (1).

With improvements in lymphoma treatment and prolonged survival, long-term complications affecting quality of life have become increasingly important. Among these complications, cancer-related cognitive impairment (CRCI) has emerged as a growing clinical concern in cancer survivors, including patients with lymphoma (3–5). CRCI manifests as declines in attention, executive function, memory, and information processing speed, significantly impairing patients’ quality of life. CRCI is a multifactorial condition and may be associated with treatment-related neurotoxicity, systemic inflammation, fatigue, emotional distress, aging, and reduced cognitive reserve (5–8). In patients with non-CNS DLBCL, the absence of direct CNS lesions makes early identification of CRCI particularly challenging, because cognitive symptoms may be subtle and conventional structural imaging may fail to detect early functional or metabolic alterations.Our preliminary study found that the baseline non-CNS DLBCL group exhibited glucose hypometabolism in multiple brain regions compared to the healthy control group. Neuropsychological scales, including the Montreal Cognitive Assessment (MoCA) and the Functional Assessment of Cancer Therapy-Cognitive Function (FACT-Cog), currently serve as practical tools for CRCI identification (5–9). However, scale-based assessment may be influenced by education, mood, fatigue, and patient-reported perception, and may not fully reflect underlying brain metabolic or structural changes.

Recent neuroimaging studies using fMRI, DTI, and FDG-PET have provided objective approaches for detecting functional, structural, and metabolic brain alterations associated with cognitive impairment (10–14).Among these modalities, ^18^F-FDG PET is particularly relevant because it quantifies regional cerebral glucose metabolism, which is closely related to neuronal activity and neurovascular coupling. Therefore, FDG-PET may provide objective metabolic information complementary to scale-based cognitive assessment (3, 8, 15). However, most studies use single- or dual-modality approaches, which lead to suboptimal detection efficiency. Advances in medical data integration and computational power have made artificial intelligence (AI)-based medical image analysis an active area of research (16). In radiomics-based workflows, quantitative features are first extracted from predefined regions of interest and then combined with clinical variables as structured tabular inputs for prediction models. Tabular deep learning models are capable of capturing nonlinear interactions and high-order feature relationships among these handcrafted imaging features and clinical variables. Importantly, this strategy differs from end-to-end image-based deep learning because raw PET/CT images are not directly input into the network (16–19). This PyRadiomics feature-based tabular modeling approach has not yet been explored in CRCI research involving DLBCL.

Therefore, the contribution of the present study should be interpreted in a specific methodological and clinical context: the focus on non-CNS DLBCL, the integration of PET-derived, CT-derived, and clinical tabular features, the comparison of multiple tabular deep learning architectures, and the use of a SHAP-based interpretability framework (3, 20–23). Accordingly, this study leveraged ^18^F-FDG PET/CT images to extract PyRadiomics features from automatically segmented brain regions and integrated these imaging-derived features with clinical variables to construct structured PET/CT tabular datasets. We developed and compared tabular deep learning models to identify patients with non-CNS DLBCL who are at risk of CRCI at an early stage, thereby providing a foundation for improving their quality of life.

## 2 Materials and methods

### 2.1 Study participants

This retrospective study enrolled non-CNS DLBCL patients (baseline or post-chemotherapy) who underwent cerebral ^18^F-FDG PET imaging at our institution between October 2020 and January 2025. Patients were further categorized according to treatment status at the time of PET/CT and cognitive assessment as either baseline/pre-treatment or post-chemotherapy. For post-chemotherapy patients, treatment regimen, and the interval from the last chemotherapy cycle to cognitive assessment were extracted from medical records when available. All cognitive assessments were completed within 48 hours before PET/CT examination. Demographic data (sex, age, years of education) were collected. Treatment-related information, including treatment status, chemotherapy regimen, and the interval from the last chemotherapy cycle to cognitive assessment, was extracted from medical records when available. The treatment-related characteristics are summarized in the Results section. All participants provided written informed consent, and the study protocol was approved by the Institutional Review Board of our institution (Ethics Approval No: KYLL-2023-0035).

**Figure 1:**
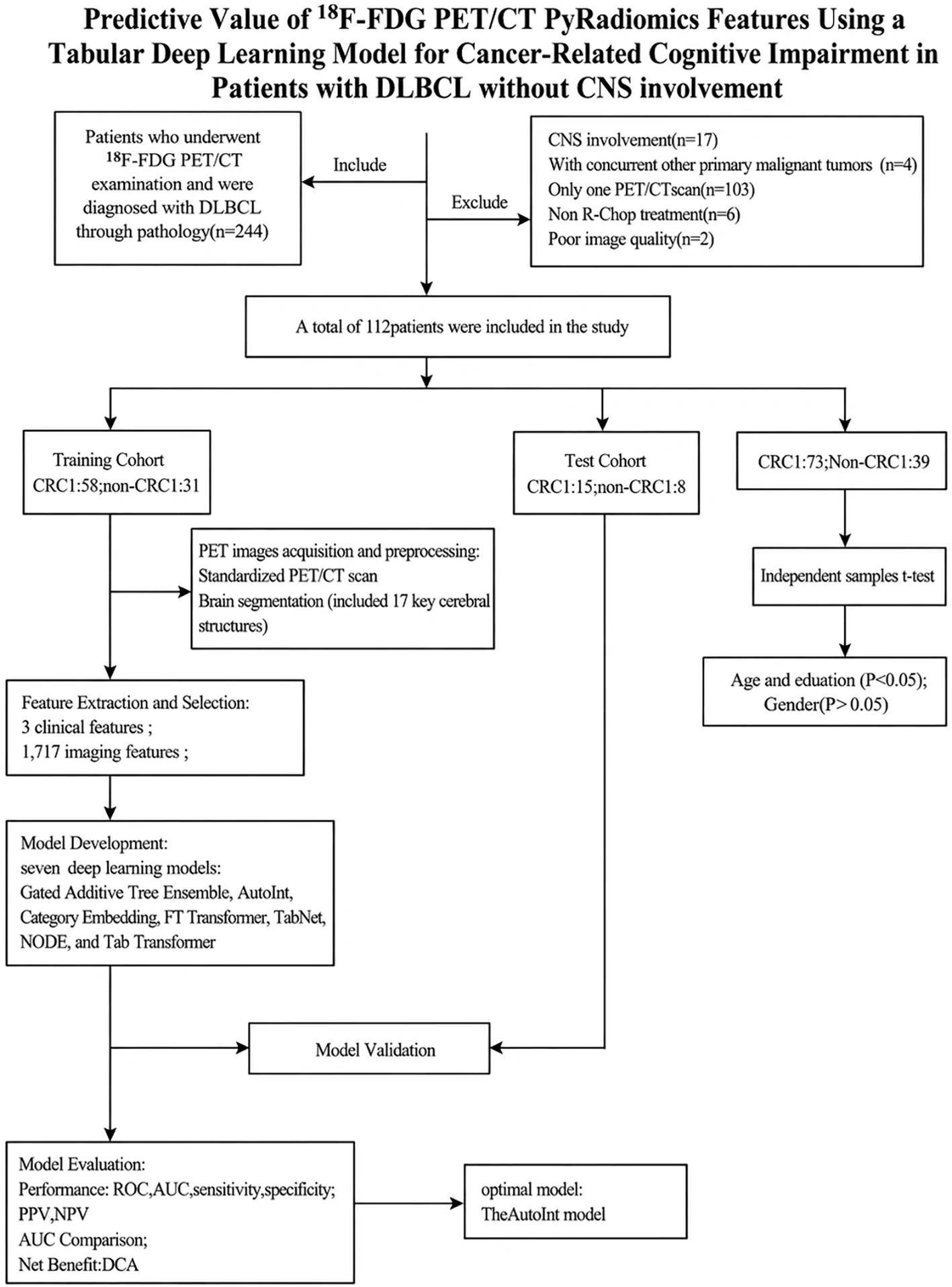
Detailed flowchart of the study process. Non-CNS DLBCL, diffuse large B-cell lymphoma without central nervous system involvement; CRCI, cancer-related cognitive impairment; NPV, negative predictive value; PPV, positive predictive value.

Inclusion criteria: (1) Age ≥18 years; (2) DLBCL group: Pathologically confirmed DLBCL without evidence of brain metastasis or other intracranial abnormalities on imaging, and completion of cerebral ^18^F-FDG PET examination. The exclusion criteria were as follows: (1) history of other malignancies; (2) pre-existing neurological or psychiatric disorders, psychological conditions, alcohol or drug abuse, severe cardiovascular or cerebrovascular disease, hyperthyroidism, diabetes mellitus, or other metabolic diseases; (3) incomplete clinical data or inability to undergo long-term follow-up; and (4) PET images with significant artifacts or defects. These exclusion criteria were applied to reduce potential CRCI mimics, including neurological, psychiatric, vascular, endocrine, metabolic, or substance-related conditions that could independently affect cognitive performance or brain FDG uptake. The research workflow is shown in **Figure 1**.

### 2.2 Cognitive assessment

The Functional Assessment of Cancer Therapy-Cognitive Function (FACT-Cog) scale comprises 37 items scored on a 0–4 Likert scale, with 28 reverse-scored items such that higher total scores reflect better cognitive functioning (9). Standardized administrator training ensured protocol adherence, and assessments were conducted in sound-attenuated booths (duration: 15–20 minutes). The total score ranges from 0 to 148, with lower scores indicating cognitive impairment; In this study, we used the Perceived Cognitive Impairment (PCI) subscale with a cut-off of < 50 (corresponding to a total score ≤ 60) to identify clinically significant cognitive decline. Separately, the Montreal Cognitive Assessment (MoCA) evaluates global cognition with a maximum score of 30. A score ≥26 denotes normal cognition, with one bonus point applied for ≤12 years of education to correct for educational bias. In the present study, CRCI was defined using a combined criterion: patients were classified as having CRCI only when they simultaneously met both of the following conditions: a MoCA score < 26 and a FACT-Cog PCI subscale score < 50, which corresponds to a total FACT-Cog score ≤ 60. Patients with an abnormal result on only one of the two scales were defined as having “subclinical cognitive decline” and were included in the non-CRCI group.

### 2.3 PET image acquisition and preprocessing

**Figure 2:**
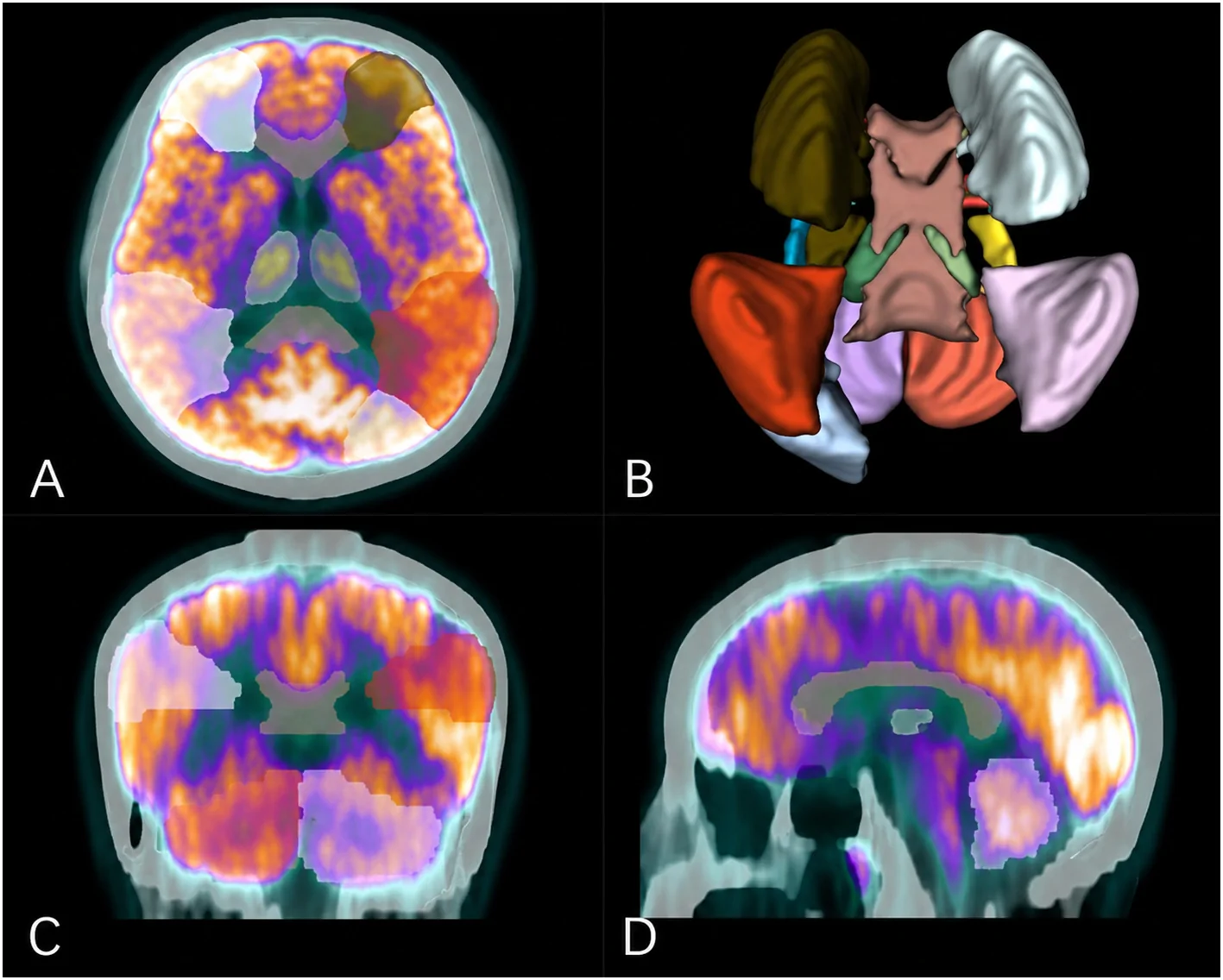
Schematic diagram of brain region segmentation. **(A)** Axial view; **(B)** 3D brain region rendering; **(C)** Coronal view; **(D)** Sagittal view. Images were generated based on the Hammersmith atlas template.

Imaging was performed using a GE Discovery VCT 64 PET/CT scanner, with ^18^F-FDG radiochemical purity >95%. Patients fasted for ≥6 h with blood glucose maintained at 90–160 mg/dL. Following intravenous ^18^F-FDG injection (3.70–5.55 MBq/kg), patients rested for 45 minutes before scanning. To minimize motion artifacts during scanning, patients’ heads were securely immobilized using a molded headrest and a Velcro restraining band upon positioning in the scanner. All brain PET data were acquired in a three-dimensional (3D) mode with a distinct brain scan, with an average acquisition duration of 10 minutes. PET images were reconstructed using a conventional iterative approach (128×128, 28 iteration subsets, 2 iterations). The head was scanned using a spiral CT protocol (120 kV; 90 mA) with a slice thickness of 5 mm and a pitch of 0.516:1, covering a volume from the skull base to the vertex. Brain segmentation constitutes a crucial step in radiomics feature extraction and structured data construction. In this study, automated brain segmentation of PET/CT images was performed using the open-source MOOSE120 software (https://github.com/QIMP-Team/MOOSE). The regions of interest (ROI) included 17 key cerebral structures. The segmentation was performed using a pre-trained automated model based on the Hammersmith atlas template (**Figure 2**). Additional preprocessing details are provided in the **Supplementary Methods**.

### 2.4 Feature extraction and selection

We extracted 101 radiomic features from PET/CT Volume of Interest (VOI) across 17 brain regions using the open-source PyRadiomics package (17). Features with a correlation coefficient threshold greater than 0.7 were selected, resulting in 377 features that were ultimately chosen for the model. These features included 19 histogram features, 26 shape features, 24 Gray-Level Co-occurrence Matrix (GLCM) features, 16 Gray-Level Run-Length Matrix (GLRLM) features, and 16 Gray-Level Size Zone Matrix (GLSZM) features. To avoid data leakage, patients were first randomly partitioned into training (n=89) and test (n=23) sets at an 8:2 ratio using stratified sampling before feature standardization or feature selection. All preprocessing steps that required estimation from data were fitted exclusively on the training set. Specifically, Z-score standardization parameters (mean and standard deviation) were calculated from the training set and then applied unchanged to the test set. Correlation-based feature selection was also performed only within the training set; the selected feature subset was then applied to the test set. This procedure yielded 377 retained features for model development. The test set was kept completely independent and was used only for final model evaluation.

### 2.5 Model development and evaluation

We developed and compared seven tabular deep learning models, including GATE, AutoInt, Category Embedding, FT Transformer, TabNet, NODE, and Tab Transformer. The models were trained using structured tabular inputs derived from PyRadiomics features and clinical variables, rather than raw PET/CT images. A stratified 8:2 split was used to generate the training set and independent test set while preserving the CRCI/non-CRCI class distribution. To improve reproducibility, a fixed random seed of 1 was used. Missing or abnormal continuous values were imputed using the median, and continuous variables were standardized using Z-score normalization. No artificial oversampling or undersampling was performed. To reduce data leakage, all preprocessing and feature selection procedures were fitted only within the training data and then applied to the test data. Five-fold cross-validation was performed within the training set for model development. The independent test set was not involved in cross-validation, feature selection, or model tuning, and was used only for final evaluation. The models were trained using the Adam optimizer with automatic learning-rate search, a maximum of 1000 epochs, a batch size of 128, and early stopping. A probability threshold of 0.5 was used for binary classification. Model performance was evaluated mainly using AUC, with sensitivity, specificity, and DCA used as secondary evaluation metrics. Because no independent external cohort was available, external validation was not performed.

### 2.6 Statistical methods

All modeling procedures and statistical analyses were performed using Python 3.9 and R software (version 4.2). The normality of continuous variables was evaluated using the Kolmogorov-Smirnov (K-S) test. Demographic characteristics between groups were compared using independent two-sample t-tests, with a two-tailed *P*-value <0.05 considered statistically significant. The diagnostic performance of different models were then comprehensively evaluated and compared based on the AUC values. Through systematic training and testing of the models, we predicted whether each patient had CRCI and calculated the sensitivity, specificity, positive predictive value (PPV), negative predictive value (NPV), and AUC for each model in both the training and test sets. To quantify uncertainty in the primary performance metric, patient-level bootstrap resampling with 2,000 iterations was used to estimate 95% confidence intervals for AUC. Sensitivity, specificity, PPV, and NPV were calculated at the predefined probability threshold of 0.5 and are presented as point estimates.

## 3 Results

### 3.1 Demographic characteristics and clinical scales

This study included 112 patients with non-CNS DLBCL. Among them, 53 patients were assessed at baseline before chemotherapy and 59 patients were assessed after chemotherapy. Among the post-chemotherapy patients, 27 received the R-CHOP regimen and 32 received the CHOP regimen. The median interval from the last chemotherapy cycle to cognitive assessment was 28 days (interquartile range, 21–42 days). All patients underwent cerebral ^18^F-FDG PET/CT examinations. Patients were stratified into two groups of CRCI (n=73) and non-CRCI (n=39), based on neuropsychological scale results. The baseline characteristics of the two patient groups are shown in **Table 1**. The mean age of patients in the CRCI group was 62 years, compared to 57 years in the non-CRCI group, with no statistically significant difference between the two groups (*P* = 0.083). Similarly, no significant differences were observed in gender distribution (*P* = 0.061) or mean years of education (*P* = 0.147) between the groups.

**Table 1:** Demographic characteristics comparison between CRCI and non-CRCI groups.

Variable	CRCI (n=73)	non-CRCI (n=39)	*P* Values
Age (years)	62.01 ± 13.01	57.33 ± 13.91	0.083
Gender (M/F)	21/52	17/22	0.061
Education (years)	6.63 ± 4.52	7.06 ± 4.78	0.147

CRCI, cancer-related cognitive impairment; non-CRCI, DLBCL patients without cancer related cognitive impairment; *P < 0.05.

### 3.2 Feature selection

We extracted radiomic features from CT and PET images of each subject across 17 predefined brain regions (including the temporal lobe, cerebellum, frontal lobe, parietal lobe, thalamus, corpus callosum, etc.). Feature extraction was performed using the PyRadiomics framework, encompassing shape, first-order statistics, and texture features (GLCM, GLDM, GLRLM, GLSZM, NGTDM). Following initial screening (e.g., a correlation coefficient threshold >0.7), the total number of features ultimately included in the analysis was 237 for the CT modality and 140 for the PET modality. The specific number and names of features retained for each brain region and corresponding modality are detailed in **Supplementary Table 1**.

### 3.3 Comparative predictive performance of seven tabular deep learning models in the training and test sets

Five-fold cross-validation within the training set was used during model development to improve the stability of model training and feature selection, while the final diagnostic performance was evaluated on the independent test set. In the comprehensive evaluation of seven tabular deep learning classifier models trained on structured PyRadiomics and clinical features for diagnosing CRCI, the performance metrics across both training and test sets are summarized in **Table 2**. Within the training set, most models exhibited strong discriminatory abilities, with AUC values ranging from 0.813 to 0.923. Notably, the Category Embedding Model achieved the highest AUC of 0.923, along with high sensitivity (0.948) and NPV (0.870). The NODE Model demonstrated the highest sensitivity (0.983) and NPV (0.950), while the GATE and Tab Transformer Models exhibited relatively higher specificity (0.774 and 0.742, respectively). In contrast, the TabNet Model performed poorly in the training set, with the lowest specificity (0.097) and AUC (0.555).

**Table 2:** Diagnostic performance of the seven tabular deep learning classifier models for CRCI.

Classifier models	Dataset	AUC (95%CI)	Sensitivity	Specificity	PPV	NPV
Gated Additive Tree Ensemble	training	0.899(0.836–0.954)	0.914	0.774	0.883	0.828
	test	0.783 (0.589–0.931)	0.800	0.500	0.750	0.571
AutoInt Model	training	0.905 (0.848– 0.955)	0.931	0.613	0.818	0.826
	test	0.858 (0.705–0.971)	0.933	0.605	0.737	0.750
Category Embedding Model	training	0.923 (0.865–0.971)	0.948	0.645	0.833	0.870
	test	0.767 (0.575– 0.929)	0.867	0.625	0.812	0.714
FT Transformer Model	training	0.820 (0.731– 0.900)	0.948	0.419	0.753	0.812
	test	0.725 (0.520– 0.895)	0.933	0.500	0.778	0.800
TabNet Model	training	0.555 (0.443–0.667)	0.914	0.097	0.654	0.375
	test	0.333 (0.133– 0.548)	0.867	0.000	0.619	0.000
NODE Model	training	0.904 (0.841–0.957)	0.983	0.613	0.826	0.950
	test	0.808 (0.611– 0.967)	0.933	0.625	0.824	0.833
Tab Transformer Model	training	0.813 (0.725–0.886)	0.810	0.742	0.855	0.676
	test	0.650 (0.439– 0.839)	0.867	0.250	0.684	0.500

AUC, Area under the curve; CRCI, cancer-related cognitive impairment; NPV, Negative predictive value; PPV, Positive predictive value.

**Figure 3:**
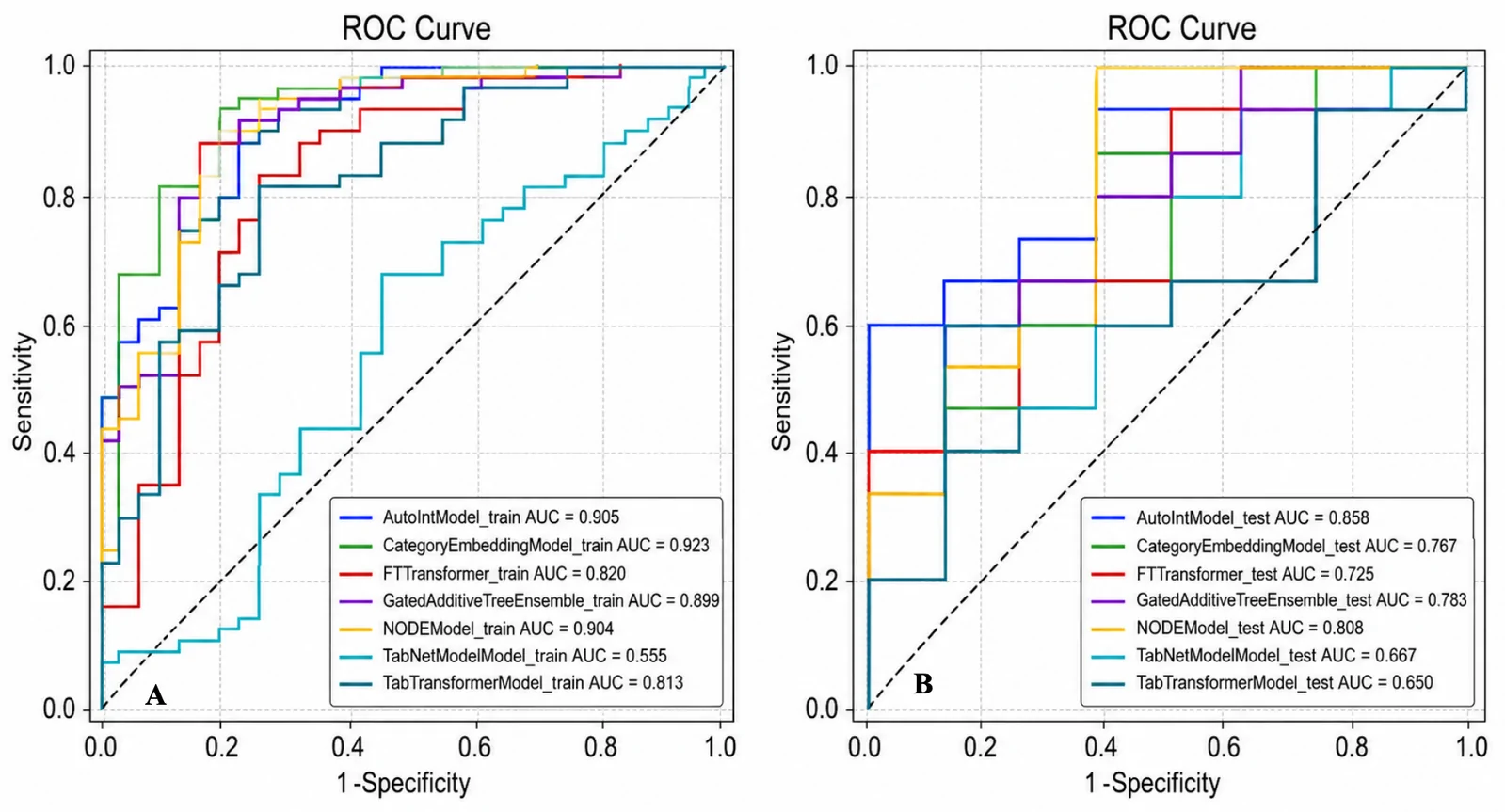
Comparative ROC curves of all models on the training and test set. **(A)** Receiver operating characteristic (ROC) curves of the seven models on the training set; **(B)** ROC curves of the seven models on the test set;seven tabular deep learning models included: Gated Additive Tree Ensemble (GATE), AutoInt, Category Embedding, FT Transformer, Transformer-based Tabular Network (TabNet), Neural Oblivious Decision Ensembles (NODE) and Tab Transformer.

When evaluated on the test set, the AutoInt model achieved the highest AUC (0.858), with a sensitivity of 0.933 and a specificity of 0.605. The NODE and Category Embedding Models also maintained reasonable performance in the test set, with AUCs of 0.808 and 0.767, respectively (**Figure 3**). However, a noticeable decline in specificity and PPV was observed for several models in the test set, particularly for TabNet, which failed to generalize effectively, yielding an AUC of only 0.333.

**Figure 4:**
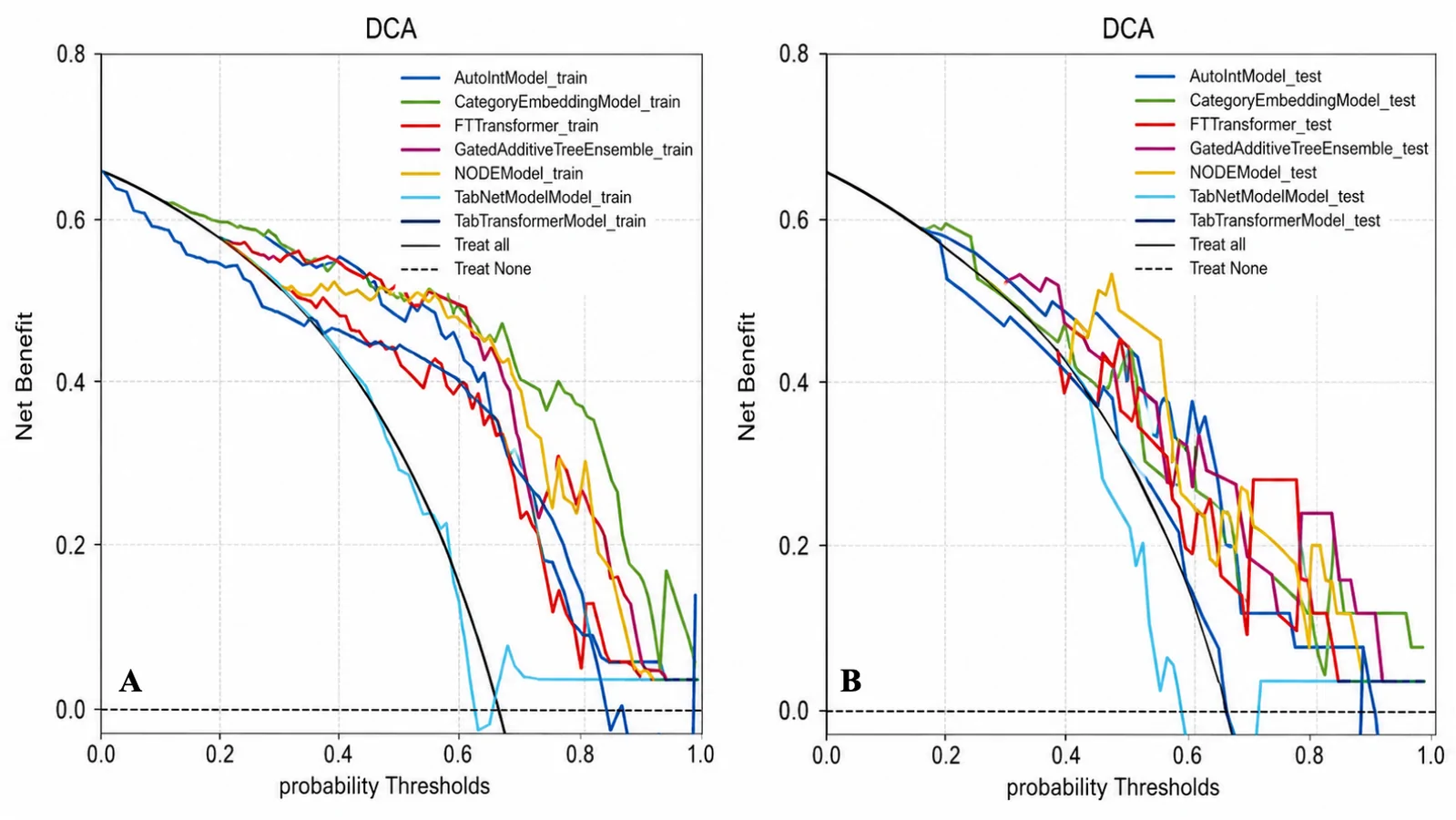
Comparison of decision curves among the seven tabular deep learning models. **(A)** Decision curves of the seven models on the training set; **(B)** Decision curves of the seven models on the test set. The y-axis represents net benefit, quantifying the clinical utility of treatment strategies. The black solid line (“Treat None”) and dashed gray line (“Treat All”) denote the net benefits of treating no patients versus all patients, respectively. Other curves represent model-specific net benefits. The left and right panels display results for the training and test set, respectively.

DCA further showed that the AutoInt and NODE models were positioned above the “Treat All” and “Treat None” lines across a clinically relevant threshold probability range (**Figure 4**). Based on these results, the AutoInt and NODE models were selected as optimal.

### 3.4 Modeling with different data modalities

**Figure 5:**
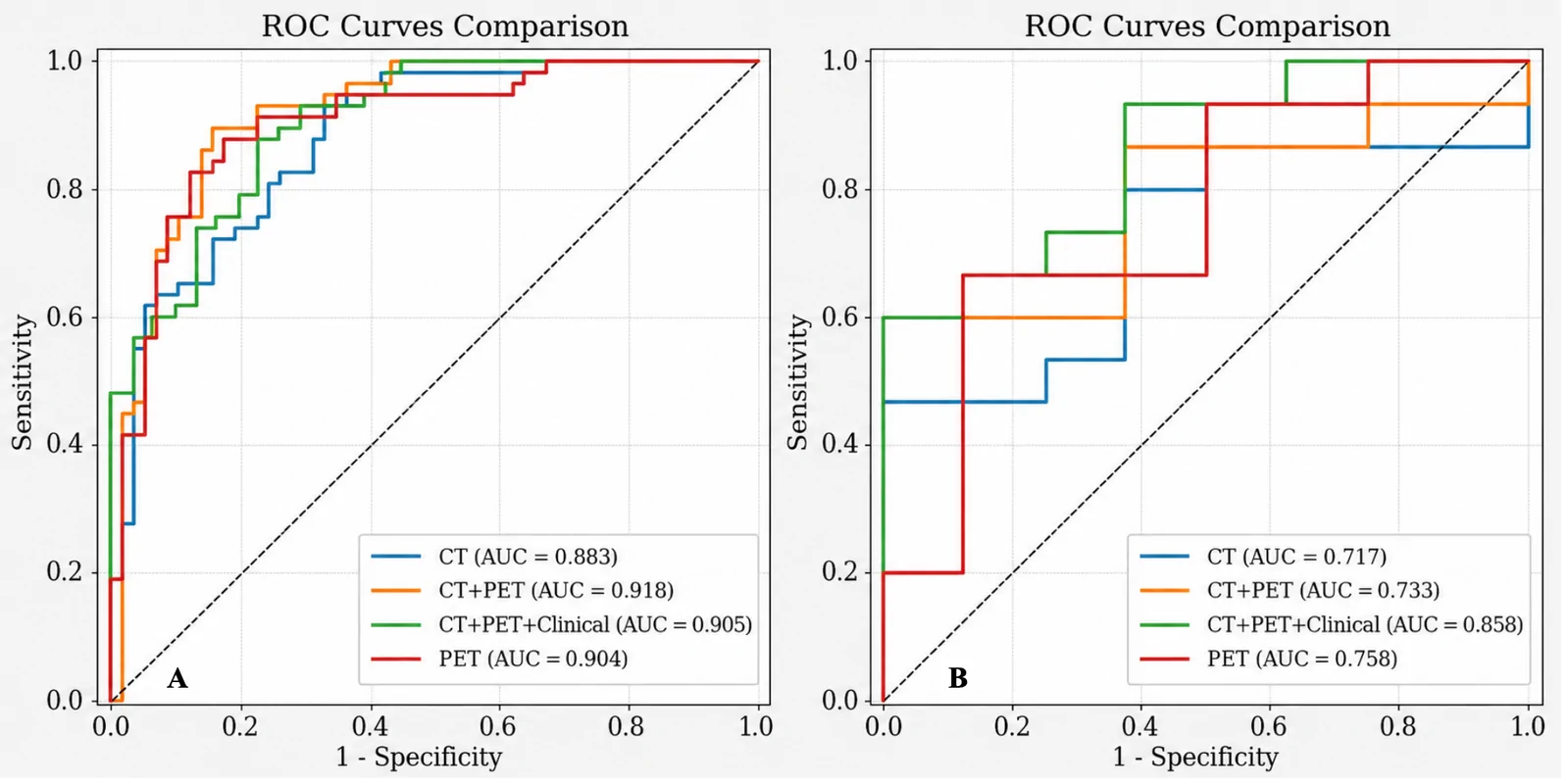
Comparison of ROC curves of the AutoInt Model on the training and test set. **(A)** Receiver operating characteristic (ROC) curves of the AutoInt Model on the training set; **(B)** ROC curves of the AutoInt Model on the test set.

**Figure 6:**
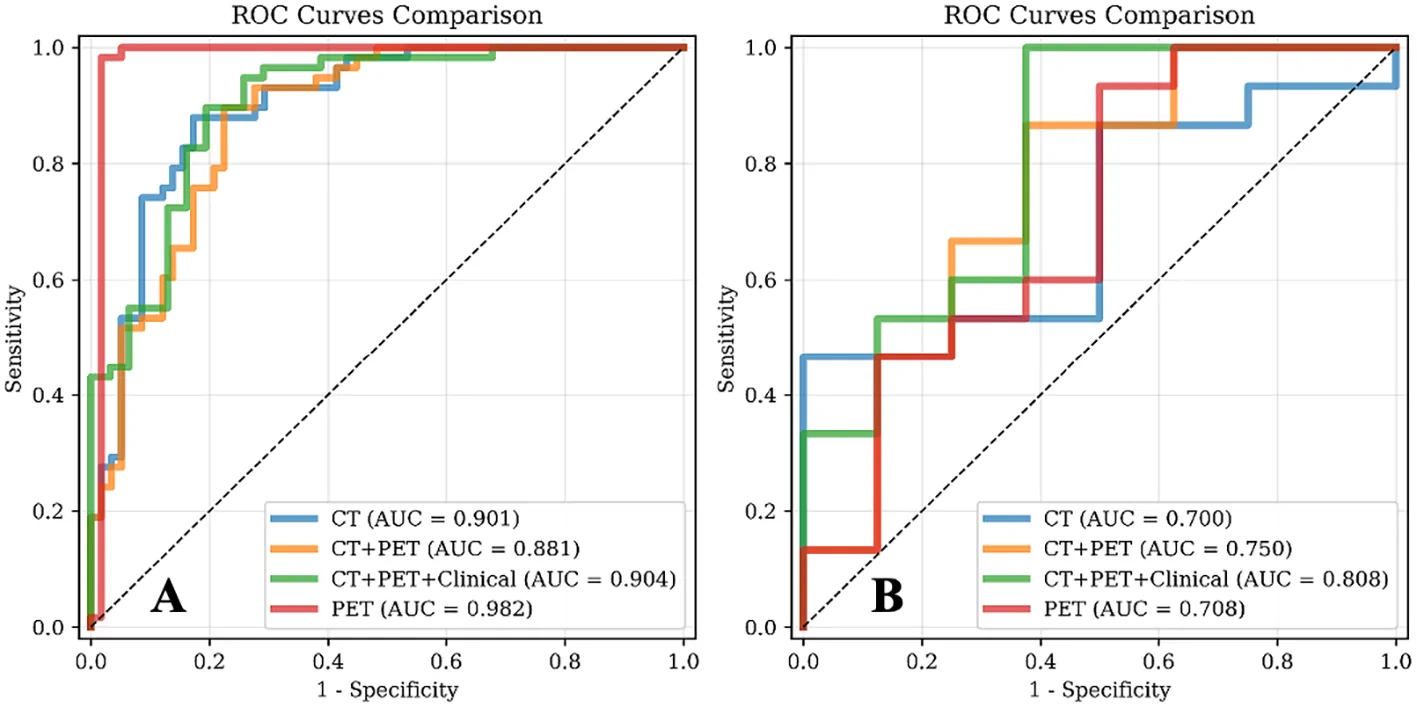
Comparison of ROC curves of the NODE Model on the training and test set. **(A)** Receiver operating characteristic (ROC) curves of the NODE Model on the training set; **(B)** ROC curves of the NODE Model on the test set.

When different data modalities were used (CT, PET, CT+PET, and CT+PET+Clinical), the two models showed different diagnostic performance (**Tables 3**, **4**). However, between the two models applied to different modalities, AutoInt achieved the highest diagnostic performance on the combined CT+PET+Clinical data, with an AUC of 0.905 (training set) and 0.858 (test set) (**Figures 5**, **6**).

**Table 3:** Diagnostic performance of the AutoInt model for CRCI in different data modalities.

Different data modalities	Dataset	AUC (95%CI)	Sensitivity	Specificity	PPV	NPV
CT	training	0.883 (0.830–0.933)	0.190	0.983	0.917	1.000
	test	0.717 (0.515– 0.892)	0.067	1.000	0.750	0.571
PET	training	0.904 (0.856– 0.949)	0.931	0.655	0.730	0.905
	test	0.758 (0.446– 0.867)	0.800	0.500	0.750	0.571
CT+PET	training	0.918 (0.871– 0.959)	0.190	0.983	0.917	0.548
	test	0.733 (0.530– 0.908)	0.067	1.000	1.000	0.364
CT+PET+Clinical	training	0.905 (0.848– 0.955)	0.931	0.613	0.818	0.826
	test	0.858 (0.705–0.971)	0.933	0.605	0.737	0.750

AUC, Area under the curve; CRCI, cancer-related cognitive impairment; NPV, Negative predictive value; PPV, Positive predictive value.

**Table 4:** Diagnostic performance of the NODE model for CRCI in different data modalities.

Different data modalities	Dataset	AUC (95%CI)	Sensitivity	Specificity	PPV	NPV
CT	training	0.901 (0.849–0.946)	0.655	0.914	0.884	0.726
	test	0.700(0.487– 0.868)	0.400	1.000	1.000	0.471
PET	training	0.982 (0.951–1.000)	0.983	0.948	0.950	0.982
	test	0.708 (0.485–0.900)	0.600	0.625	0.750	0.455
CT+PET	training	0.881 (0.825– 0.930)	0.879	0.776	0.797	0.865
	test	0.750 (0.529–0.930)	0.667	0.750	0.833	0.545
CT+PET+Clinical	training	0.904 (0.841–0.957)	0.983	0.613	0.826	0.950
	test	0.808 (0.611– 0.967)	0.933	0.625	0.824	0.833

AUC, Area under the curve; CRCI, cancer-related cognitive impairment; NPV, Negative predictive value; PPV, Positive predictive value; NODE, Neural Oblivious Decision Ensembles.

### 3.5 Ranked contributions of multi-modal brain regional features to the prediction model

**Figure 7:**
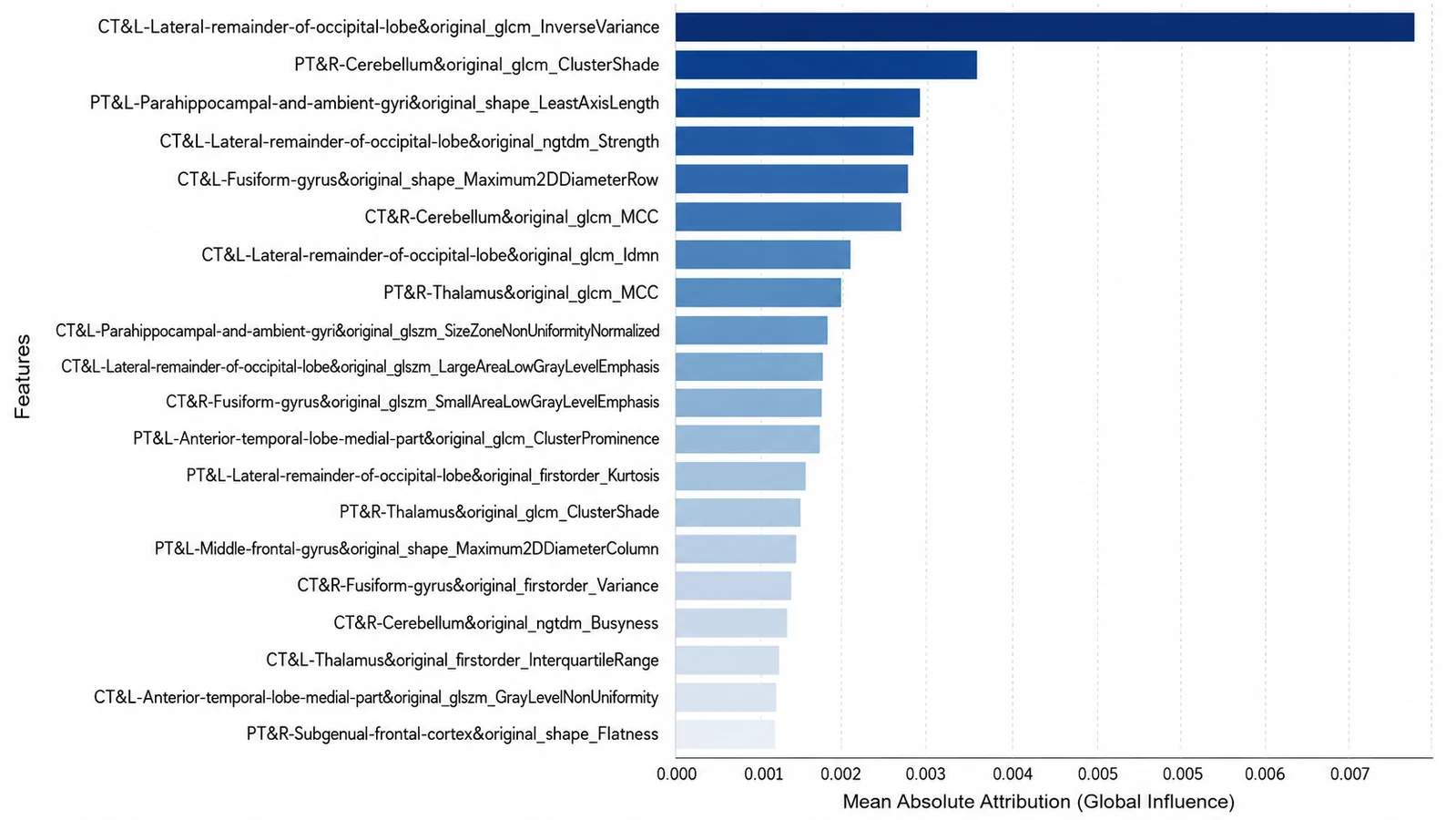
Ranked contributions of multi-modal brain regional features to the prediction model.

In this study, the global contribution of individual radiomic features to the prediction model was evaluated using SHAP (SHapley Additive exPlanations) values, with the resulting feature importance ranking presented in **Figure 7**. Our SHAP analysis identified CT&L-Lateral-remainder-of-occipital-lobe&original_glcm_InverseVariance as the highest-ranked feature in this cohort. However, because SHAP-based feature ranking does not establish biological causality or biomarker validity, this feature was interpreted as an exploratory imaging-derived predictor rather than a validated biomarker. The most influential features originated from both CT and PET imaging modalities and were distributed across multiple brain regions, including the occipital lobe, cerebellum, parahippocampal gyrus, fusiform gyrus, thalamus, and frontal lobe. These high-importance features spanned several categories: texture features (e.g., glcm_InverseVariance, glcm_ClusterShade, ngtdm_Strength), shape features (e.g., shape_LeastAxisLength, shape_Maximum2D DiameterRow), and first-order statistical features (e.g., firstorder_Kurtosis, firstorder_Variance).

## 4 Discussion

In this study, we developed and evaluated a multimodal PyRadiomics feature-based tabular deep learning framework for predicting CRCI in patients with non-CNS DLBCL. The main findings were as follows. First, tabular deep learning models trained on handcrafted PET/CT PyRadiomics features and clinical variables showed preliminary predictive potential for CRCI prediction. Second, among the seven evaluated models, AutoInt achieved the best test-set performance, suggesting that attention-based feature interaction learning may be useful for structured radiomics and clinical data. Third, the integration of PET-derived, CT-derived, and clinical features achieved the highest test-set AUC among the evaluated AutoInt input modalities, supporting the potential complementary value of multimodal information. Finally, SHAP analysis provided an interpretable ranking of model-contributing features, although these features should be regarded as exploratory rather than validated biomarkers.

Previous studies have demonstrated the significant value of ^18^F-FDG PET in the cognitive assessment of neurodegenerative diseases (15). however, its application in the context of cognitive impairment associated with DLBCL remains unexplored. Our preliminary research has revealed persistent glucose hypometabolism in multiple brain regions of non-CNS DLBCL patients, which correlates with the degree of cognitive impairment. Nevertheless, traditional visual assessment methods for PET imaging exhibit considerable subjectivity, making it challenging to detect subtle metabolic changes. This limitation aligns with the findings of Verma RK. in their research on Alzheimer’s disease (24). In recent years, AI has revolutionized medical image analysis (25, 26). However, it is important to clarify that the present study did not employ end-to-end convolutional or image-based deep learning. Instead, after automated brain segmentation, we extracted handcrafted PET/CT PyRadiomics features and combined them with clinical variables as structured tabular inputs (17, 18). The tabular deep learning models used in this study were designed to learn nonlinear relationships among these extracted features rather than to learn image representations directly from raw PET/CT voxels (19). Existing CRCI studies remain predominantly confined to single- or dual-modality data analyses (3, 10–14), and effective integration of PET-derived, CT-derived, and clinical tabular information is still lacking. The present study therefore proposes a multimodal PyRadiomics feature-based tabular deep learning framework for predicting CRCI in patients with non-CNS DLBCL.

Building on previous FDG-PET studies of CRCI in DLBCL and PET radiomics studies of cognitive impairment, the present study further explored whether PET/CT PyRadiomics features combined with clinical variables could support CRCI prediction in a non-CNS DLBCL cohort. In terms of model architecture selection, we systematically compared seven state-of-the-art tabular modeling approaches: TabNet, NODE, TabTransformer, FTTransformer, AutoInt, GATE, along with a baseline Category Embedding model. The AutoInt model achieved the highest test-set AUC among the evaluated tabular models. This may be related to its self-attention mechanism, which is designed to learn high-order feature interactions and may be suitable for high-dimensional structured inputs such as radiomics features. This finding is consistent with previous studies showing that structured medical data are often high-dimensional, heterogeneous, and sparse, and that specially designed tabular deep learning architectures using feature embedding, attention mechanisms, and explicit feature-interaction modules are capable of capturing nonlinear and high-order relationships in tabular inputs (27–29). In the modality-specific analysis, the PET-only NODE model showed a high training AUC of 0.982, but its test AUC decreased to 0.708, suggesting potential overfitting and highlighting the importance of model generalizability in medical AI evaluation (30). It should also be noted that data leakage was minimized in our workflow by separating the training and test sets before feature standardization and feature selection. Standardization parameters and correlation-based feature selection were fitted only within the training set and then applied to the independent test set. Nevertheless, because the number of radiomic features was large relative to the sample size, overfitting remains an important concern and external validation is still required.

Although the Category Embedding model achieved the highest training AUC of 0.923, its test AUC decreased to 0.767, suggesting potential overfitting. TabNet enhances precision and interpretability through dynamic selection of key clinical indicators. However, its sensitivity to training data volume restricts applicability in small-scale studies, this sensitivity to training data volume may have contributed to the suboptimal performance observed in our cohort (AUC = 0.555). This result is consistent with previous evidence that model performance estimates in small-sample radiomics studies may be unstable and highly dependent on the training-test split (31). In this study, Tab Transformer and FT Transformer exhibited distinct performance profiles, which is consistent with previous observations that deep learning models for tabular data may show dataset-dependent performance, especially in small-sample or heterogeneous datasets (32). In contrast, the Tab Transformer showed relatively balanced performance on the training set but suffered from a marked degradation in specificity (from 0.742 to 0.250) and severe overfitting on the test set. Studies have demonstrated that it excels in multi-disease prediction tasks (33). Ultimately, both models achieved suboptimal overall generalization, with AUCs of 0.725 and 0.650, respectively, underscoring the persistent challenges in directly deploying such architecturally complex models in clinical scenarios characterized by limited sample sizes. The GATE model demonstrated strong performance in the training set, achieving high overall diagnostic efficacy (AUC = 0.899) with a well-balanced sensitivity (0.914) and specificity (0.774), alongside excellent positive (0.883) and negative (0.828) predictive values. However, its generalization capability noticeably declined in the test set: specificity substantially dropped to 0.500 and the negative predictive value decreased to 0.571, although sensitivity remained relatively high at 0.800. The test set AUC decreased to 0.783, indicating a tendency of overfitting. Nevertheless, compared to other complex models (e.g., Tab Transformer and FT Transformer), the performance degradation was relatively controlled, and the model maintained moderate discriminative ability in the test set (34). Given our study’s focus on diagnostic efficacy prediction with similarly constrained data, this may partly explain the relatively limited performance degradation observed for GATE in this cohort.

Our study also evaluated four modeling strategies based on different data modalities: CT, PET, CT+PET, and CT+PET+Clinical. At the data modality level, individual imaging techniques exhibited inherent and complementary limitations. The CT model has high specificity, which makes it reliable for excluding non-cases. However, its low sensitivity results in a high rate of missed diagnoses, limiting its effectiveness as a standalone screening tool (33). In contrast, the PET model showed relatively higher sensitivity but lower specificity in the present cohort (35). The integration of these two modalities significantly enhanced model performance. Most notably, the CT+PET+Clinical model achieved the highest test-set AUC among the evaluated AutoInt input modalities, with an AUC of 0.858. This finding suggests that PET-derived metabolic features, CT-derived texture features, and clinical variables may provide complementary information for CRCI prediction. PET features may reflect regional metabolic heterogeneity, CT texture features may capture subtle structural or attenuation-related differences, and clinical variables such as age and education may provide patient-level context related to cognitive vulnerability and cognitive reserve (6, 7, 36).

By incorporating clinical variables together with imaging-derived PyRadiomics features, the model may capture complementary patient-level information beyond imaging characteristics alone (36). Beyond modality-level analysis, at the model architecture level, our findings illuminate the hazards associated with an overzealous pursuit of accuracy within the training set. The NODE model exhibited severe overfitting in multimodal tabular modeling, as evidenced by a dramatic drop in test set performance following its near-flawless training AUC of 0.982. This finding emphasizes that in the medical field a model’s capacity for generalization serves as a more vital evaluation criterion than its performance on the training set (30). Therefore, further validation is required before the model can be considered for future clinical application.

The clinical variables included in this study, particularly age and years of education, were selected because age is a well-established factor associated with cognitive decline, whereas educational attainment is commonly used as a proxy measure of cognitive reserve (6, 7). Although patients with CRCI tended to be older and had slightly fewer years of education than those without CRCI, these differences did not reach statistical significance. Therefore, these demographic patterns should be interpreted cautiously. Nevertheless, age and education were retained as clinically relevant variables because age is associated with cognitive decline, whereas education is commonly used as a proxy measure of cognitive reserve (6–8). This observed pattern not only aligns with predictions derived from the cognitive reserve theory but is also consistent with findings reported by Carroll et al. in their investigation of cognitive function among cancer survivors (8). Although the limited sample size of the test set may have constrained statistical power, the consistent manifestation of this trend across both training and test cohorts lends further support to the relevance of the cognitive reserve theory in elucidating the mechanisms underlying CRCI.

Based on SHAP interpretability analysis, the most important predictor in our model was the inverse variance texture feature derived from CT images of the left lateral remainder of the occipital lobe. In radiomics, inverse variance—a gray-level co-occurrence matrix (GLCM) feature—quantifies local homogeneity; a higher value indicates more uniform tissue texture (37). The prominence of this feature should be interpreted with caution. CRCI is generally considered to involve distributed cognitive networks, including frontal, temporal, hippocampal, and attention-related circuits; therefore, an occipital CT texture feature is not necessarily expected to represent a primary CRCI-specific signal. In a small single-center retrospective cohort, such a feature may also be influenced by partial-volume effects, reconstruction-related texture variation, or segmentation boundary effects (38, 39). As a hub for integrating visual and multisensory information, the structural integrity of the occipital lobe may be influenced by or interact with disease processes in connected brain regions, such as the temporal or frontal lobes, through large-scale neural networks. Furthermore, our study employed a multimodal (CT and PET) and multi-regional feature analysis approach encompassing regions including the cerebellum, parahippocampal gyrus, thalamus, and frontal lobe. These results suggest that multimodal and multi-regional features may provide broader information for CRCI prediction (40). Accordingly, this feature should be regarded as an exploratory model-derived imaging feature rather than a validated biomarker. Further studies with external validation and formal robustness testing across reconstruction protocols and segmentation perturbations are required before assigning biological or clinical significance to this finding.

Building on the PET/CT radiomics and clinical tabular framework used in this study, future CRCI prediction may be extended toward broader multimodal inference. In cancer-related cognitive assessment, PET/CT-derived imaging features could be integrated with histopathological information, longitudinal neuropsychological profiles, and wearable device-derived digital biomarkers, including physical activity, sleep quality, and daily behavioral patterns. Recent studies in older adults have shown that multimodal behavioral markers and accelerometer-derived activity patterns can provide complementary information for cognitive and physical function assessment (41–44). These non-invasive longitudinal data may help overcome the temporal limitations of imaging and clinical variables, thereby improving model flexibility, interpretability, and real-world applicability. In addition, foundation models pretrained on large-scale multimodal medical data may offer a promising approach for integrating imaging, clinical, pathological, and wearable-derived information. Future prospective studies should explore multimodal foundation-model-based frameworks for CRCI prediction and individualized risk stratification.

However, several limitations should be acknowledged. This was a single-center retrospective study with a relatively small sample size and no external validation cohort. Although five-fold cross-validation and bootstrap-based AUC confidence intervals were added, the high feature dimensionality relative to the sample size may still increase the risk of overfitting, and calibration performance was not fully evaluated. In addition, formal radiomics robustness analysis, including ICC analysis and segmentation perturbation testing, was not performed. The available clinical variables were limited, and conventional machine-learning models were not included as baseline comparators. Future prospective multicenter studies with external validation, calibration assessment, radiomics robustness testing, expanded clinical-biological variables, and conventional machine-learning comparisons are needed.

## 5 Conclusions

Tabular deep learning models based on handcrafted PET/CT PyRadiomics features showed preliminary potential for predicting CRCI in patients with non-CNS DLBCL. Among the evaluated models, AutoInt achieved the most favorable performance in the exploratory internal test set. Multimodal integration of PET, CT, and clinical tabular features may provide complementary information for CRCI prediction. SHAP analysis identified a left occipital CT texture feature as the top-ranked model contributor; however, this finding should be regarded as exploratory rather than a validated biomarker.

## Data Availability

The raw data supporting the conclusions of this article will be made available by the authors, without undue reservation.
