# Supplementary material for: Predictive value of 18F-FDG PET/CT PyRadiomics features using a tabular deep learning model for cancer-related cognitive impairment in patients with DLBCL without CNS involvement
Source: Front Oncol. 2026 Aug 12;16:1861773. doi: 10.3389/fonc.2026.1861773 (PMC13506380; doi:10.3389/fonc.2026.1861773)
Supplement: Supplementary file 1 [file Table1.docx]

Supplementary Material

# 1 Supplementary Tables

**Supplementary Table 1 The specific number and names of features.**

| Image Acquisition | Region | feature count | feature names |
| --- | --- | --- | --- |
| CT | Right anterior medial temporal lobe | 19 | shape_Maximum2DDiameterRow |
|  |  |  | shape_SurfaceArea |
|  |  |  | firstorder_Kurtosis |
|  |  |  | firstorder_Minimum |
|  |  |  | firstorder_Variance |
|  |  |  | glcm_Imc1 |
|  |  |  | glcm_InverseVariance |
|  |  |  | gldm_DependenceNonUniformityNormalized |
|  |  |  | gldm_SmallDependenceLowGrayLevelEmphasis |
|  |  |  | glrlm_LongRunLowGrayLevelEmphasis |
|  |  |  | glszm_GrayLevelNonUniformity |
|  |  |  | glszm_LargeAreaLowGrayLevelEmphasis |
|  |  |  | glszm_LowGrayLevelZoneEmphasis |
|  |  |  | glszm_SizeZoneNonUniformityNormalized |
|  |  |  | glszm_SmallAreaEmphasis |
|  |  |  | glszm_SmallAreaLowGrayLevelEmphasis |
|  |  |  | ngtdm_Busyness |
|  |  |  | ngtdm_Coarseness |
|  |  |  | ngtdm_Strength |
|  | Left anterior medial temporal lobe | 17 | shape_LeastAxisLength |
|  |  |  | firstorder_Kurtosis |
|  |  |  | firstorder_Minimum |
|  |  |  | firstorder_Variance |
|  |  |  | glcm_Idmn |
|  |  |  | glcm_Imc1 |
|  |  |  | glcm_InverseVariance |
|  |  |  | gldm_DependenceNonUniformityNormalized |
|  |  |  | gldm_LargeDependenceLowGrayLevelEmphasis |
|  |  |  | gldm_SmallDependenceLowGrayLevelEmphasis |
|  |  |  | glszm_GrayLevelNonUniformity |
|  |  |  | glszm_GrayLevelNonUniformityNormalized |
|  |  |  | glszm_SizeZoneNonUniformityNormalized |
|  |  |  | glszm_SmallAreaEmphasis |
|  |  |  | glszm_SmallAreaLowGrayLevelEmphasis |
|  |  |  | ngtdm_Busyness |
|  |  |  | ngtdm_Coarseness |
|  | Left parahippocampal gyrus | 20 | shape_MeshVolume |
|  |  |  | firstorder_Kurtosis |
|  |  |  | firstorder_Minimum |
|  |  |  | firstorder_Variance |
|  |  |  | glcm_Correlation |
|  |  |  | glcm_Imc1 |
|  |  |  | glcm_MCC |
|  |  |  | gldm_DependenceNonUniformity |
|  |  |  | gldm_DependenceNonUniformityNormalized |
|  |  |  | gldm_DependenceVariance |
|  |  |  | gldm_LargeDependenceEmphasis |
|  |  |  | gldm_LargeDependenceLowGrayLevelEmphasis |
|  |  |  | glrlm_RunLengthNonUniformity |
|  |  |  | glszm_GrayLevelNonUniformity |
|  |  |  | glszm_SizeZoneNonUniformityNormalized |
|  |  |  | glszm_SmallAreaEmphasis |
|  |  |  | glszm_SmallAreaLowGrayLevelEmphasis |
|  |  |  | glszm_ZoneEntropy |
|  |  |  | ngtdm_Busyness |
|  |  |  | ngtdm_Coarseness |
|  | right fusiform gyrus | 14 | shape_SurfaceArea |
|  |  |  | firstorder_Kurtosis |
|  |  |  | firstorder_Variance |
|  |  |  | glcm_Idmn |
|  |  |  | glcm_InverseVariance |
|  |  |  | gldm_DependenceNonUniformityNormalized |
|  |  |  | gldm_DependenceVariance |
|  |  |  | glrlm_LongRunLowGrayLevelEmphasis |
|  |  |  | glszm_LargeAreaHighGrayLevelEmphasis |
|  |  |  | glszm_SizeZoneNonUniformityNormalized |
|  |  |  | glszm_SmallAreaEmphasis |
|  |  |  | glszm_SmallAreaLowGrayLevelEmphasis |
|  |  |  | glszm_ZoneVariance |
|  |  |  | ngtdm_Coarseness |
|  | Left fusiform gyrus | 18 | shape_Flatness |
|  |  |  | shape_Maximum2DDiameterRow |
|  |  |  | firstorder_Kurtosis |
|  |  |  | firstorder_Minimum |
|  |  |  | firstorder_Variance |
|  |  |  | glcm_Correlation |
|  |  |  | glcm_Idmn |
|  |  |  | glcm_InverseVariance |
|  |  |  | gldm_DependenceNonUniformityNormalized |
|  |  |  | gldm_DependenceVariance |
|  |  |  | gldm_SmallDependenceLowGrayLevelEmphasis |
|  |  |  | glrlm_LongRunLowGrayLevelEmphasis |
|  |  |  | glszm_GrayLevelNonUniformity |
|  |  |  | glszm_LargeAreaHighGrayLevelEmphasis |
|  |  |  | glszm_SmallAreaEmphasis |
|  |  |  | glszm_SmallAreaLowGrayLevelEmphasis |
|  |  |  | glszm_ZoneVariance |
|  |  |  | ngtdm_Busyness |
|  | right cerebellum | 18 | shape_MajorAxisLength |
|  |  |  | shape_Maximum2DDiameterRow |
|  |  |  | shape_Maximum2DDiameterSlice |
|  |  |  | shape_SurfaceArea |
|  |  |  | firstorder_Kurtosis |
|  |  |  | firstorder_Minimum |
|  |  |  | firstorder_Variance |
|  |  |  | glcm_Correlation |
|  |  |  | glcm_Idmn |
|  |  |  | glcm_Imc1 |
|  |  |  | glcm_InverseVariance |
|  |  |  | glcm_MCC |
|  |  |  | gldm_DependenceNonUniformityNormalized |
|  |  |  | glszm_SizeZoneNonUniformityNormalized |
|  |  |  | glszm_SmallAreaLowGrayLevelEmphasis |
|  |  |  | ngtdm_Busyness |
|  |  |  | ngtdm_Coarseness |
|  |  |  | ngtdm_Strength |
|  | Left cerebellum | 14 | shape_LeastAxisLength |
|  |  |  | shape_Maximum2DDiameterRow |
|  |  |  | firstorder_Kurtosis |
|  |  |  | firstorder_Minimum |
|  |  |  | glcm_Correlation |
|  |  |  | glcm_Idmn |
|  |  |  | glcm_Imc1 |
|  |  |  | glcm_InverseVariance |
|  |  |  | glcm_MCC |
|  |  |  | gldm_SmallDependenceLowGrayLevelEmphasis |
|  |  |  | glszm_LargeAreaHighGrayLevelEmphasis |
|  |  |  | glszm_LowGrayLevelZoneEmphasis |
|  |  |  | glszm_SizeZoneNonUniformityNormalized |
|  |  |  | ngtdm_Coarseness |
|  | left lateral occipital remainder | 15 | shape_LeastAxisLength |
|  |  |  | shape_MajorAxisLength |
|  |  |  | firstorder_Kurtosis |
|  |  |  | firstorder_Minimum |
|  |  |  | firstorder_Variance |
|  |  |  | glcm_Correlation |
|  |  |  | glcm_Idmn |
|  |  |  | glcm_Imc1 |
|  |  |  | glcm_InverseVariance |
|  |  |  | glcm_MCC |
|  |  |  | glrlm_LongRunLowGrayLevelEmphasis |
|  |  |  | glszm_GrayLevelNonUniformity |
|  |  |  | glszm_LargeAreaLowGrayLevelEmphasis |
|  |  |  | glszm_SmallAreaLowGrayLevelEmphasis |
|  |  |  | ngtdm_Strength |
|  | left middle frontal gyrus | 8 | firstorder_Kurtosis |
|  |  |  | glcm_Correlation |
|  |  |  | glcm_MCC |
|  |  |  | gldm_DependenceVariance |
|  |  |  | glrlm_LongRunLowGrayLevelEmphasis |
|  |  |  | glszm_GrayLevelNonUniformity |
|  |  |  | glszm_SmallAreaEmphasis |
|  |  |  | ngtdm_Strength |
|  | Right middle frontal gyrus | 8 | shape_Flatness |
|  |  |  | firstorder_Kurtosis |
|  |  |  | firstorder_Variance |
|  |  |  | glcm_InverseVariance |
|  |  |  | glrlm_LongRunLowGrayLevelEmphasis |
|  |  |  | glszm_GrayLevelNonUniformity |
|  |  |  | glszm_SizeZoneNonUniformityNormalized |
|  |  |  | glszm_SmallAreaLowGrayLevelEmphasis |
|  | left inferolateral parietal remainder | 14 | shape_LeastAxisLength |
|  |  |  | shape_Maximum2DDiameterRow |
|  |  |  | shape_MinorAxisLength |
|  |  |  | firstorder_Kurtosis |
|  |  |  | firstorder_Range |
|  |  |  | glcm_Correlation |
|  |  |  | glcm_Imc1 |
|  |  |  | glcm_MCC |
|  |  |  | gldm_SmallDependenceLowGrayLevelEmphasis |
|  |  |  | glrlm_LongRunLowGrayLevelEmphasis |
|  |  |  | glszm_GrayLevelNonUniformity |
|  |  |  | glszm_LargeAreaLowGrayLevelEmphasis |
|  |  |  | glszm_SmallAreaEmphasis |
|  |  |  | ngtdm_Strength |
|  | Right inferolateral parietal remainder | 12 | shape_LeastAxisLength |
|  |  |  | shape_MeshVolume |
|  |  |  | firstorder_Range |
|  |  |  | glcm_Correlation |
|  |  |  | glcm_Idmn |
|  |  |  | glcm_MCC |
|  |  |  | glrlm_LongRunLowGrayLevelEmphasis |
|  |  |  | glszm_GrayLevelNonUniformity |
|  |  |  | glszm_LargeAreaLowGrayLevelEmphasis |
|  |  |  | glszm_SizeZoneNonUniformityNormalized |
|  |  |  | ngtdm_Busyness |
|  |  |  | ngtdm_Strength |
|  | left thalamus | 13 | shape_Elongation |
|  |  |  | shape_LeastAxisLength |
|  |  |  | shape_Maximum2DDiameterRow |
|  |  |  | shape_SurfaceArea |
|  |  |  | firstorder_InterquartileRange |
|  |  |  | glcm_Correlation |
|  |  |  | gldm_DependenceNonUniformityNormalized |
|  |  |  | gldm_DependenceVariance |
|  |  |  | glszm_GrayLevelNonUniformity |
|  |  |  | glszm_LargeAreaLowGrayLevelEmphasis |
|  |  |  | glszm_SizeZoneNonUniformityNormalized |
|  |  |  | glszm_SmallAreaLowGrayLevelEmphasis |
|  |  |  | ngtdm_Coarseness |
|  | Right thalamus | 12 | shape_Elongation |
|  |  |  | shape_Flatness |
|  |  |  | shape_LeastAxisLength |
|  |  |  | shape_MeshVolume |
|  |  |  | firstorder_TotalEnergy |
|  |  |  | glcm_Correlation |
|  |  |  | gldm_DependenceNonUniformityNormalized |
|  |  |  | gldm_SmallDependenceLowGrayLevelEmphasis |
|  |  |  | glszm_GrayLevelNonUniformity |
|  |  |  | glszm_LargeAreaLowGrayLevelEmphasis |
|  |  |  | glszm_SizeZoneNonUniformityNormalized |
|  |  |  | glszm_SmallAreaLowGrayLevelEmphasis |
|  | corpus callosum | 16 | shape_MajorAxisLength |
|  |  |  | shape_Maximum2DDiameterColumn |
|  |  |  | shape_Maximum2DDiameterSlice |
|  |  |  | shape_Sphericity |
|  |  |  | shape_VoxelVolume |
|  |  |  | firstorder_Kurtosis |
|  |  |  | firstorder_Range |
|  |  |  | firstorder_Skewness |
|  |  |  | glcm_Imc1 |
|  |  |  | gldm_DependenceNonUniformityNormalized |
|  |  |  | gldm_DependenceVariance |
|  |  |  | glszm_GrayLevelNonUniformity |
|  |  |  | glszm_SmallAreaEmphasis |
|  |  |  | glszm_ZoneVariance |
|  |  |  | ngtdm_Busyness |
|  |  |  | ngtdm_Coarseness |
|  | left subgenual frontal cortex | 11 | shape_Elongation |
|  |  |  | shape_Maximum2DDiameterRow |
|  |  |  | shape_MinorAxisLength |
|  |  |  | shape_SurfaceArea |
|  |  |  | firstorder_Kurtosis |
|  |  |  | glcm_MCC |
|  |  |  | gldm_DependenceNonUniformityNormalized |
|  |  |  | gldm_DependenceVariance |
|  |  |  | glszm_GrayLevelNonUniformity |
|  |  |  | glszm_SmallAreaLowGrayLevelEmphasis |
|  |  |  | ngtdm_Busyness |
|  | Right subgenual frontal cortex | 12 | shape_Elongation |
|  |  |  | shape_Maximum2DDiameterColumn |
|  |  |  | shape_Maximum2DDiameterRow |
|  |  |  | shape_MinorAxisLength |
|  |  |  | firstorder_InterquartileRange |
|  |  |  | firstorder_Kurtosis |
|  |  |  | glcm_MCC |
|  |  |  | gldm_DependenceNonUniformityNormalized |
|  |  |  | gldm_DependenceVariance |
|  |  |  | gldm_SmallDependenceLowGrayLevelEmphasis |
|  |  |  | glszm_GrayLevelNonUniformity |
|  |  |  | glszm_SmallAreaLowGrayLevelEmphasis |
| PET | right anteromedial temporal lobe | 10 | shape_LeastAxisLength |
|  |  |  | firstorder_Skewness |
|  |  |  | glcm_ClusterShade |
|  |  |  | glcm_Correlation |
|  |  |  | glcm_Idmn |
|  |  |  | glcm_Imc1 |
|  |  |  | glcm_InverseVariance |
|  |  |  | gldm_SmallDependenceLowGrayLevelEmphasis |
|  |  |  | glszm_LargeAreaHighGrayLevelEmphasis |
|  |  |  | glszm_SmallAreaEmphasis |
|  | Left anteromedial temporal lobe | 9 | glcm_ClusterProminence |
|  |  |  | glcm_ClusterShade |
|  |  |  | glcm_Correlation |
|  |  |  | glcm_Idmn |
|  |  |  | glcm_Imc1 |
|  |  |  | glcm_MCC |
|  |  |  | gldm_SmallDependenceLowGrayLevelEmphasis |
|  |  |  | glszm_LargeAreaHighGrayLevelEmphasis |
|  |  |  | glszm_SmallAreaEmphasis |
|  | left parahippocampal gyrus | 10 | shape_LeastAxisLength |
|  |  |  | firstorder_Kurtosis |
|  |  |  | firstorder_Skewness |
|  |  |  | glcm_ClusterShade |
|  |  |  | glcm_Contrast |
|  |  |  | glcm_Idmn |
|  |  |  | glcm_Imc1 |
|  |  |  | glcm_MCC |
|  |  |  | gldm_DependenceNonUniformityNormalized |
|  |  |  | gldm_SmallDependenceLowGrayLevelEmphasis |
|  | right fusiform gyrus | 7 | shape_Elongation |
|  |  |  | glcm_ClusterProminence |
|  |  |  | glcm_ClusterShade |
|  |  |  | glcm_Correlation |
|  |  |  | glcm_Idmn |
|  |  |  | glcm_MCC |
|  |  |  | glszm_LargeAreaHighGrayLevelEmphasis |
|  | Left fusiform gyrus | 9 | shape_Elongation |
|  |  |  | glcm_ClusterShade |
|  |  |  | glcm_Correlation |
|  |  |  | glcm_Idmn |
|  |  |  | glcm_MCC |
|  |  |  | gldm_SmallDependenceLowGrayLevelEmphasis |
|  |  |  | glszm_LargeAreaHighGrayLevelEmphasis |
|  |  |  | glszm_SmallAreaEmphasis |
|  |  |  | ngtdm_Coarseness |
|  | right cerebellum | 5 | glcm_ClusterShade |
|  |  |  | glcm_Correlation |
|  |  |  | glcm_Idmn |
|  |  |  | glcm_MCC |
|  |  |  | glszm_SmallAreaEmphasis |
|  | left cerebellum | 5 | shape_Maximum3DDiameter |
|  |  |  | shape_MeshVolume |
|  |  |  | glcm_Idmn |
|  |  |  | glcm_Imc1 |
|  |  |  | glcm_MCC |
|  | left lateral occipital remainder | 7 | shape_Maximum2DDiameterRow |
|  |  |  | firstorder_Kurtosis |
|  |  |  | firstorder_Skewness |
|  |  |  | glcm_ClusterShade |
|  |  |  | glcm_Correlation |
|  |  |  | glcm_Idmn |
|  |  |  | glcm_MCC |
|  | left middle frontal gyrus | 6 | shape_Flatness |
|  |  |  | shape_LeastAxisLength |
|  |  |  | shape_Maximum2DDiameterColumn |
|  |  |  | shape_Maximum2DDiameterSlice |
|  |  |  | glcm_Idmn |
|  |  |  | glcm_MCC |
|  | right middle frontal gyrus | 8 | shape_LeastAxisLength |
|  |  |  | shape_Maximum2DDiameterRow |
|  |  |  | glcm_Idmn |
|  |  |  | glcm_Imc1 |
|  |  |  | glcm_MCC |
|  |  |  | gldm_SmallDependenceLowGrayLevelEmphasis |
|  |  |  | ngtdm_Coarseness |
|  |  |  | ngtdm_Strength |
|  | left inferolateral parietal remainder | 4 | glcm_Idmn |
|  |  |  | glcm_MCC |
|  |  |  | gldm_SmallDependenceLowGrayLevelEmphasis |
|  |  |  | glszm_SmallAreaEmphasis |
|  | right inferolateral parietal remainder | 5 | glcm_Correlation |
|  |  |  | glcm_Idmn |
|  |  |  | glcm_MCC |
|  |  |  | gldm_SmallDependenceLowGrayLevelEmphasis |
|  |  |  | glszm_SmallAreaEmphasis |
|  | left thalamus | 7 | firstorder_Kurtosis |
|  |  |  | glcm_ClusterShade |
|  |  |  | glcm_Idmn |
|  |  |  | glcm_InverseVariance |
|  |  |  | gldm_LargeDependenceHighGrayLevelEmphasis |
|  |  |  | glszm_SmallAreaLowGrayLevelEmphasis |
|  |  |  | ngtdm_Coarseness |
|  | right thalamus | 9 | shape_Maximum2DDiameterRow |
|  |  |  | firstorder_Kurtosis |
|  |  |  | firstorder_Skewness |
|  |  |  | glcm_ClusterShade |
|  |  |  | glcm_Correlation |
|  |  |  | glcm_Imc1 |
|  |  |  | glcm_MCC |
|  |  |  | gldm_SmallDependenceLowGrayLevelEmphasis |
|  |  |  | ngtdm_Coarseness |
|  | corpus callosum | 12 | shape_Elongation |
|  |  |  | shape_LeastAxisLength |
|  |  |  | firstorder_Kurtosis |
|  |  |  | glcm_ClusterProminence |
|  |  |  | glcm_Correlation |
|  |  |  | glcm_Idn |
|  |  |  | glcm_Imc1 |
|  |  |  | glcm_MCC |
|  |  |  | gldm_LargeDependenceHighGrayLevelEmphasis |
|  |  |  | gldm_SmallDependenceLowGrayLevelEmphasis |
|  |  |  | glszm_GrayLevelNonUniformity |
|  |  |  | ngtdm_Coarseness |
|  | left subgenual frontal cortex | 10 | firstorder_Kurtosis |
|  |  |  | firstorder_Skewness |
|  |  |  | glcm_ClusterShade |
|  |  |  | glcm_DifferenceVariance |
|  |  |  | glcm_Idmn |
|  |  |  | glcm_MCC |
|  |  |  | gldm_DependenceNonUniformityNormalized |
|  |  |  | gldm_LargeDependenceHighGrayLevelEmphasis |
|  |  |  | gldm_SmallDependenceLowGrayLevelEmphasis |
|  |  |  | glszm_SmallAreaLowGrayLevelEmphasis |
|  | Right subgenual frontal cortex | 13 | shape_Flatness |
|  |  |  | shape_Sphericity |
|  |  |  | firstorder_Kurtosis |
|  |  |  | firstorder_Skewness |
|  |  |  | glcm_ClusterShade |
|  |  |  | glcm_Correlation |
|  |  |  | glcm_DifferenceVariance |
|  |  |  | glcm_Imc1 |
|  |  |  | glcm_InverseVariance |
|  |  |  | gldm_DependenceNonUniformityNormalized |
|  |  |  | gldm_LargeDependenceHighGrayLevelEmphasis |
|  |  |  | gldm_SmallDependenceLowGrayLevelEmphasis |
|  |  |  | glszm_SizeZoneNonUniformityNormalized |
