# Supplementary material for: Predictive value of 18F-FDG PET/CT PyRadiomics features using a tabular deep learning model for cancer-related cognitive impairment in patients with DLBCL without CNS involvement
Source: Front Oncol. 2026 Aug 12;16:1861773. doi: 10.3389/fonc.2026.1861773 (PMC13506380; doi:10.3389/fonc.2026.1861773)
Supplement: Supplementary file 2 [file Table2.docx]

****Supplementary Methods****

**Supplementary Methods: Image Preprocessing and Segmentation Quality Control**

1. **Image Resampling**

To reduce variability caused by differences in voxel size and image acquisition geometry, PET and CT images were resampled to a uniform isotropic voxel spacing before radiomics feature extraction. Specifically, PET and CT intensity images were resampled to an isotropic voxel spacing of 2 × 2 × 2 mm³ using third-order B-spline interpolation, whereas ROI masks were resampled using nearest-neighbor interpolation to preserve label integrity.

2. **Gray-Level Discretization**

Gray-level discretization was performed before radiomics texture feature extraction to reduce the influence of image noise and intensity-scale variability. For CT images, discretization was performed using a fixed-bin-width strategy. For PET images, SUV-based intensity values were discretized using the same fixed-bin-width strategy before texture feature extraction. The discretization settings were kept consistent across all subjects.

3. **PET Intensity Normalization**

PET voxel intensities were converted into standardized uptake values (SUVs) according to injected activity, body weight, and acquisition information before radiomics feature extraction. This SUV conversion was performed to improve comparability of PET-derived features across patients.

4. **CT Reconstruction Parameters**

All CT scans were acquired and reconstructed using a standardized protocol on the GE Discovery VCT 64 PET/CT system. The CT parameters were as follows: tube voltage, 120 kV; tube current, 90 mA; slice thickness, 5 mm; matrix size, 512 × 512; pitch, 0.516:1; and reconstruction algorithm, standard filtered back projection. These parameters were kept consistent across all included subjects to minimize acquisition-related variability.

5. **Segmentation Quality Control**

Brain segmentation was performed automatically using the MOOSE120 framework. To ensure segmentation accuracy and consistency, all automatically generated brain ROIs were visually reviewed before radiomics feature extraction. The quality-control review focused on anatomical boundary accuracy, ROI completeness, image-registration quality, segmentation leakage, and segmentation defects. Cases with visible segmentation errors were rechecked by an experienced nuclear medicine physician. Only ROIs passing visual quality control were included in subsequent radiomics analyses.

### 6. Feature-Level Processing

After radiomics feature extraction, feature-level preprocessing was performed for model development. Z-score standardization was applied to extracted radiomic and clinical tabular features. To avoid data leakage, standardization parameters were estimated only from the training set and then applied unchanged to the test set. Correlation-based feature selection was also performed only within the training set, and the selected feature subset was subsequently applied to the independent test set. Missing or abnormal continuous values were imputed using the median before model development.

**7. Limitation Regarding Feature Robustness**
Formal radiomics feature robustness assessment using interobserver or intraobserver ICC analysis was not performed in the present retrospective study. Therefore, radiomics feature stability against segmentation variability, reconstruction changes, and scanner differences could not be quantitatively established. This limitation has been acknowledged in the revised manuscript, and future studies should include ICC analysis, segmentation perturbation, and external validation to confirm the robustness of the identified radiomics features.
